# A Statistical Study on the Effect of Hydrostatic Pressure on Metastable Pitting Corrosion of X70 Pipeline Steel

**DOI:** 10.3390/ma10111307

**Published:** 2017-11-14

**Authors:** Zixuan Yang, Bo Kan, Jinxu Li, Yanjing Su, Lijie Qiao, Alex A. Volinsky

**Affiliations:** 1 Corrosion and Protection Center, Key Laboratory for Environmental Fracture (MOE), University of Science and Technology Beijing, Beijing 100083, China; Zixuan_Yang2017@163.com (Z.Y.); Kanbo10008@163.com (B.K.); yjsu@ustb.edu.cn (Y.S.); lqiao@ustb.edu.cn (L.Q.); 2 Department of Mechanical Engineering, University of South Florida, Tampa, FL 33620, USA; volinsky@usf.edu

**Keywords:** X70 steel, SEM, scanning kelvin probe force microscopy (SKPFM), hydrostatic pressure, potentiostatic, pitting corrosion

## Abstract

Hydrostatic pressure effects on pitting initiation and propagation in X70 steel are investigated by evaluating metastable pitting probability using electrochemical methods and immersion corrosion tests in containing chlorine ion solution. Potentiodynamic tests indicated that hydrostatic pressure can decrease the breakdown potential and lead to a reduced transpassivity region. Metastable test results revealed that hydrostatic pressure can increase metastable pitting formation frequency and promote stabilization of metastable pitting growth. Electrochemical impedance spectroscopy (EIS) results indicate that Hydrostatic pressure decreases the charge transfer resistance and increases the dissolution rate within the cavities. Corrosion test results also indicated that pitting initiation and propagation are accelerated by hydrostatic pressure. Result validity was verified by evaluating metastable pitting to predict pitting corrosion resistance.

## 1. Introduction

There are significant amounts of resources in the deep ocean, including oil, gas and valuable minerals. The deep sea is a complex environment, subjecting materials to high hydrostatic pressure. Materials face huge challenges in the deep-sea environment [1]. X70 pipeline steel is used for deep sea oil exploration and recovery. Stress corrosion cracking (SCC) and corrosion significantly effect pipeline steel life. Usually, corrosion and SCC of pipeline steel originate from pitting corrosion. Thus, it is necessary to evaluate pitting susceptibility of the X70 pipeline steel in the deep sea environment.

Pitting corrosion initiation and propagation can be affected by microstructure, chemical composition and the environment [2,3]. Hydrostatic pressure may change metal electrochemical behavior due to environmental effects [4,5,6,7,8,9,10,11]. Beccaria et al. [4,5,6,7,8] studied the effects of hydrostatic pressure on corrosion behavior of passive metals using a pressure vessel. They found that the pitting susceptibility of Ni and Al increased with hydrostatic pressure. Zhang et al. [9] reported that pitting susceptibility of stainless steel deteriorated with hydrostatic pressure. Yang et al. [10,11] reported that high hydrostatic pressure accelerated pit growth rate in Ni–Cr–Mo–V steel. While previous studies focused on passive metals, there are only a few fundamental reports of the hydrostatic pressure effects on pitting of active metals. However, the hydrostatic effects on the steel corrosion mechanism is not clear.

Pitting corrosion is characterized by the three steps of pit initiation, metastable propagation and stabilization [12]. Metastable pitting is an important factor to evaluate pitting corrosion. Many researchers have studied metastable pitting to predict pitting tendency and propagation rate [13,14,15,16,17,18,19,20]. Amin et al. [17] studied metastable pitting events in Al and found that metastable pitting potential (E_m_) is proportional to the pitting potential (E_p_). Tian et al. reported that metastable pitting lifetime, current peak value and nuclei frequency increased with increasing potential in stainless steel [3]. Gholami et al. studied grain size effects on pitting by evaluating metastable pitting events [16]. Guan et al. predicted cyclic stress effects on pitting by studying metastable pitting of stainless steel [15]. Many previous reports had focused on metastable pitting behavior of passive metals. However, X70 steel is an active metal, and pitting corrosion performance of carbon steel is likely to be completely different from passive metals [21,22].

In this work, hydrostatic pressure effects on pitting corrosion initiation and propagation of X70 pipeline steel were studied by evaluating metastable pitting events using electrochemical methods in 0.5 mol/L NaHCO_3_ + 0.1 mol/L NaCl solution. Statistical measurements of metastable pits characteristics were performed by using the potentiostatic method to obtain useful information about the pitting initiation and stabilization. To verify the potentiostatic method accuracy, immersion corrosion experiments were also performed in 0.1 mol/L NaCl solution.

## 2. Experimental Methods

### 2.1. Materials and Sample Preparation

X70 pipeline steel is used in this experiment. Chemical composition of the steel is listed in Table 1. Specimens were not heat treated before the tests.

Small specimens for potentiostatic and potentiodynamic polarization tests with 2 × 2 × 5 mm size were cut from the X70 plates. The non-working surface of the specimen was sealed with silica gel. Standard corrosion specimens (10 mm × 10 mm × 5 mm) were used for corrosion tests. Working face of the specimens was sanded by emery paper from 400 to 5000 number and then the specimens were polished with 1 μm diamond paste. Finally, the specimens were ultrasonically cleaned in ethanol [23].

### 2.2. Electrochemical Measurements during Hydrostatic Pressure Loading

Experiments were carried out in a pressure vessel, where pressure was changed, while other parameters were kept constant. Temperature was controlled by a thermostatic bath at 25 °C. Solution was kept air saturated with oxygen at 5 mg/L (ppm) concentration, which was the same at 0.1 MPa.

Electrochemical measurements were carried out in 0.5 mol/L NaHCO_3_ + 0.1 mol/L NaCl solution using Gamry electrochemical workstation (Gamry, Reference 6000, Warminster, PA, USA). The solution was prepared using analytical grade chemicals and deionized water. This solution was used because it is gentler than the 3.5% NaCl solution to investigate the corrosion process. Electrochemical experimental device was comprised of three electrodes system. The counter electrode was platinum foil and the reference electrode was Ag/AgCl electrode.

During potentiodynamic polarization measurements, the potential was scanned from −360 mv vs. Ag/AgCl electrode. Potential scanning rate was kept at 0.2 mV/s to anodic direction until stable pitting occured. Data acquisition rate was 20 Hz and no data smoothing were applied [13].

Samples were potentiostatically polarized at −100 mV vs. Ag/AgCl electrode for 2000 s and at the same time the current response was recorded at 20 Hz frequency. Current peak value of 20 nA was defined as the critical value of a metastable event. The morphology of potentiostatically polarized samples was observed by scanning electron microscopy (SEM). Each electrochemical measurement was repeated 3 times under the same conditions.

Electrochemical impedance spectroscopy (EIS) tests during immersion test were conducted at a frequency ranging from 0.01 Hz to 100 kHz with a 5 mV amplitude signal at open circuit potential.

### 2.3. Stable Pit Propagation Measurements

To verify hydrostatic pressure effects on stable pitting growth and ensure potentiostatic method validity, immersion corrosion tests were conducted in 0.1 mol/L NaCl solution at 0.1 MPa, 5 MPa and 10 MPa for 30 min, respectively. 3D profiles of typical corrosion pitting on the X70 steel surface after 30 min immersion at different hydrostatic pressure in 0.1 mol/L NaCl solution were measured by confocal scanning laser microscope (Olympus, LEXT 3100, Tokyo, Japan) [11].

### 2.4. Microstructure Characterization

The inclusions in X70 pipeline steel were observed by SEM. Chemical composition of inclusions were analyzed with energy dispersive spectra (EDS) attached to an SEM (Zeiss, EVO MA10/LS 10, Oberkochen, Germany).

## 3. Results

Our previous work had reported that there are three inclusions types in the X70 steel. Type A inclusion is complex, consisting of two parts. One part of the inclusion is rich in Mn, Ca and S, while the other part is rich in Al, Ca and O. Two parts of Type A inclusion can be clearly distinguished based on the morphology. Type B inclusion is only rich in Al, Ca and O, while type C inclusion is only rich in Mn, Ca and S [24].

### 3.1. Metastable Pitting Analysis

**Figure 1:**
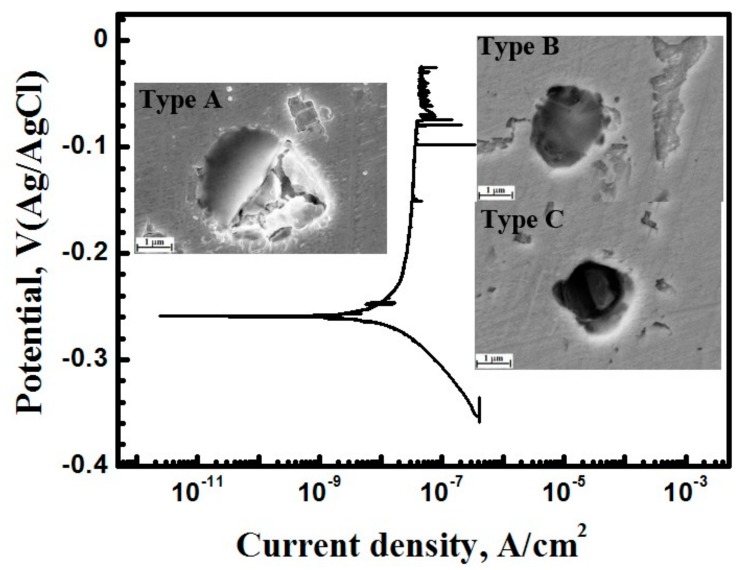
Slow scan rate potentiodynamic polarization curve and metastable pits morphology generated during the test in 0.5 mol/L NaHCO_3_ + 0.1 mol/L NaCl solution.

Previous workers had focused on metastable pitting behavior of passive metals [16,17,18]. However, we will study the metastable pitting behavior of active X70 steel. Figure 1 shows potentiodynamic polarization curves of the X70 steel obtained with 0.2 mV/s scanning rate in 0.5 mol/L NaHCO_3_ + 0.1 mol/L NaCl solution at atmospheric pressure. The potentiodynamic polarization test was stopped at the region between metastable pitting potential E_m_ and E_p_. Morphology of metastable pitting at inclusion sites after potentiodynamic polarization test was studied by SEM. Pits were clearly generated at the inclusion positions. Passive film of the type A inclusion had been broken at the site richer in Mn, Ca and S, while passive film of the type B inclusion is intact. Passive film of the type C inclusion had been broken through and the inclusion was partially dissolved. Passive film of types A and C inclusions are easier to be broken through than the metal matrix surface. This result indicated that type A and C inclusion can induce metastable pits and type B inclusion cannot induce metastable pits at the passive range of passive region. Metastable pits were generated at the active inclusions positions. Thus, type A and C is active inclusions and type B is not active inclusion [24].

### 3.2. Electrochemical Results

#### 3.2.1. Potentiodynamic Polarization Curves

**Figure 2:**
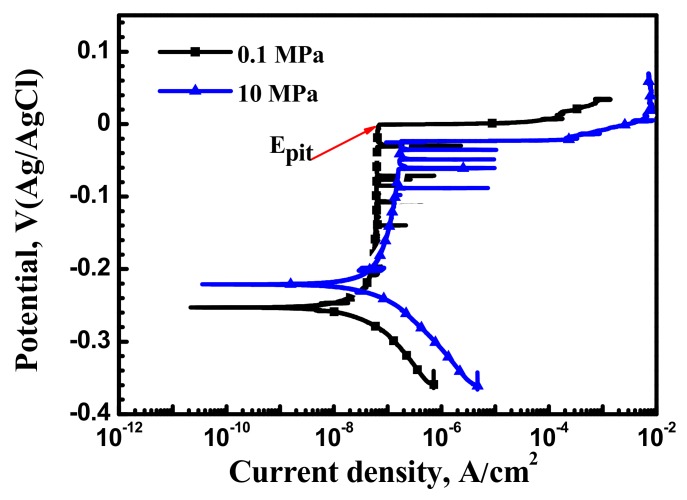
Polarization curves at 0.1 MPa and 10 MPa obtained with 0.2 mV/s scanning rate.

Potentiodynamic curves of the X70 pipeline steel at 0.1 MPa, 5 MPa and 10 MPa are shown in Figure 2. Current fluctuations were observed in the passive region during potentiodynamic test. Current fluctuations can be regarded as metastable pitting events. Figure 2 shows that the E_pit_ of 0.1 MPa at 8 mV SCE is higher than at 10 MPa (−24 mV) in the 0.5 mol/L NaHCO_3_ + 0.1 mol/L NaCl solution. Alloy pitting corrosion resistance increases with E_pit_, this means that the X70 pipeline steel corrosion resistance is worsened by hydrostatic pressure. Furthermore, Figure 2 indicates that the passivity current density slightly increases with hydrostatic pressure.

#### 3.2.2. Metastable Pitting Electrochemistry

Stable pitting can develop from metastable pitting at the early stages [25], and stable pitting corrosion product is directly related to metastable pitting characteristics [24,25,26]. Potentiostatic measurements were carried out at constant potential to evaluate pitting initiation and stabilization from metastable pitting at different hydrostatic pressure.

**Figure 3:**
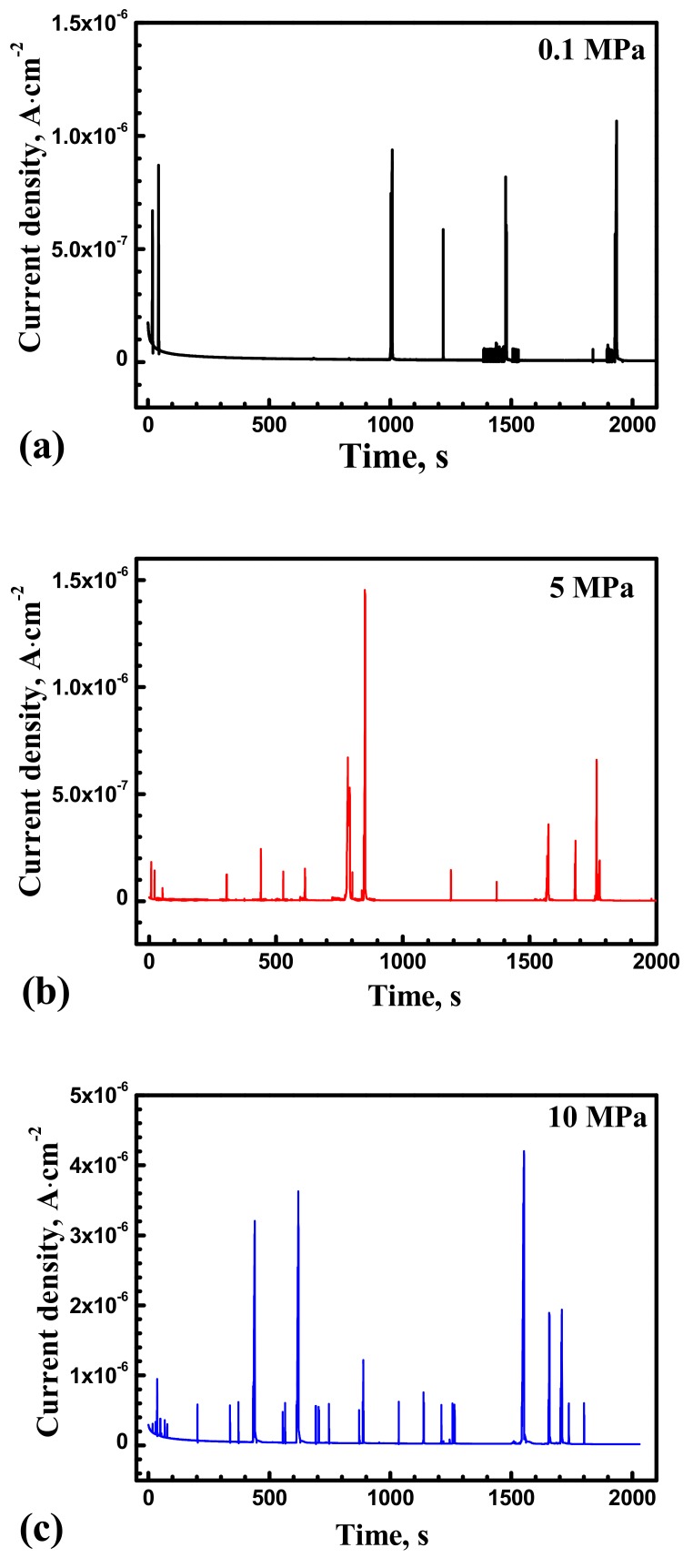
Typical current vs. time curves of the X70 steel at various hydrostatic pressure in 0.5 mol/L NaHCO_3_ + 0.1 mol/L NaCl solution obtained under −100 mV vs. Ag/AgCl electrode: (**a**) 0.1 MPa; (**b**) 5 MPa; (**c**) 10 MPa.

**Figure 4:**
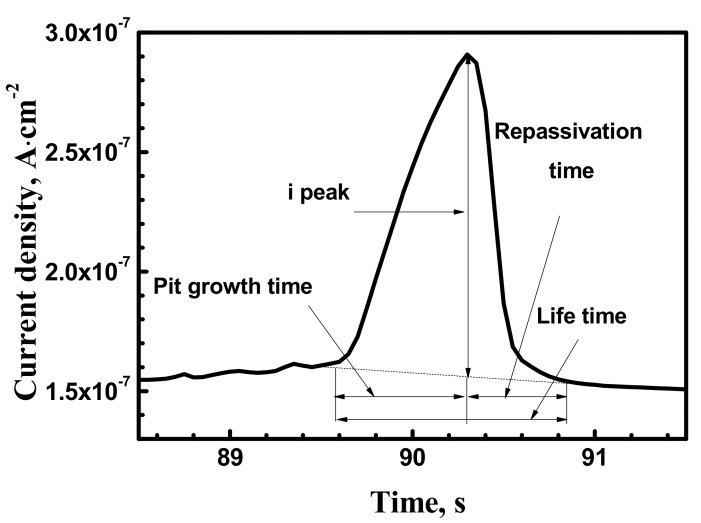
Typical shape of current transient in 0.5 mol/L and NaHCO_3_ + 0.1 mol/L NaCl solution at –100 mV vs. Ag/AgCl electrode.

Figure 3 shows time vs. current curves for the X70 steel at 0.1 MPa, 5 MPa and 10 MPa obtained with −100 mV vs. Ag/AgCl electrode. Metastable pitting parameters were obtained and analyzed by statistical methods. Figure 4 shows local magnification of Figure 3 with typical current rise due to inclusion dissolution or abrupt passive film breakdown, followed by current quick decay corresponding to newly formed passive film. Metastable current transients do not increase to a high enough value, keeping stable pit growth. Current transient peak response shows local dissolution followed by repassivation.

Hydrostatic pressure affects the shape, size and frequency of current peaks in metastable pitting. Metastable current transients at different hydrostatic pressure can be described by maximum current (i_max_), peak current (i_peak_), base current (i_bc_), pit growth time (t_g_) and repassivation time (t_rp_). Lifetime of a metastable pitting event is defined as, t_pit_ = t_g_ + t_rp_. Peak current (i_peak_) is defined as, i_peak_ = i_max_ − i_bc_ [2]. These parameters are shown in Figure 4.

**Figure 5:**
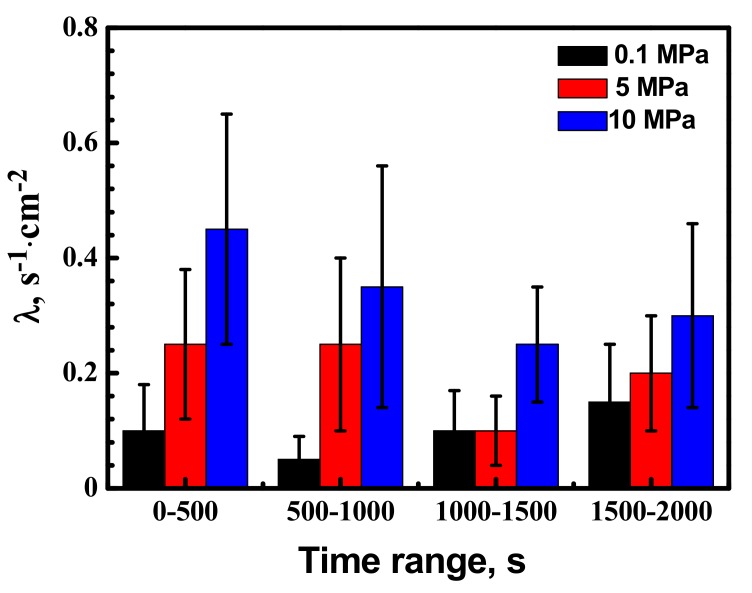
Metastable pit frequency in 0.5 mol/L NaHCO_3_ + 0.1 mol/L NaCl aqueous solution for the X70 steel at various hydrostatic pressure and −100 mV vs. Ag/AgCl electrode.

Figure 5 shows average metastable pit nucleation frequency λ (cm^−2^·s^−1^) for the X70 steel at various hydrostatic pressure with anodic potential −100 mV vs. Ag/AgCl electrode. The λ values were calculated by counting the number of events every 500 s. Higher hydrostatic pressure causes an increase in the frequency of metastable pitting events. The nucleation frequency reduced with time due to elimination of pit nucleation sites from the electrode surface. In the first 500 s, higher percentage of metastable pitting events was found. Metastable pitting frequency increase with hydrostatic pressure has been demonstrated. The result indicated that hydrostatic pressure makes many more metastable pits produce at inclusion sites. More inclusion can be activated by higher hydrostatic pressure.

**Figure 6:**
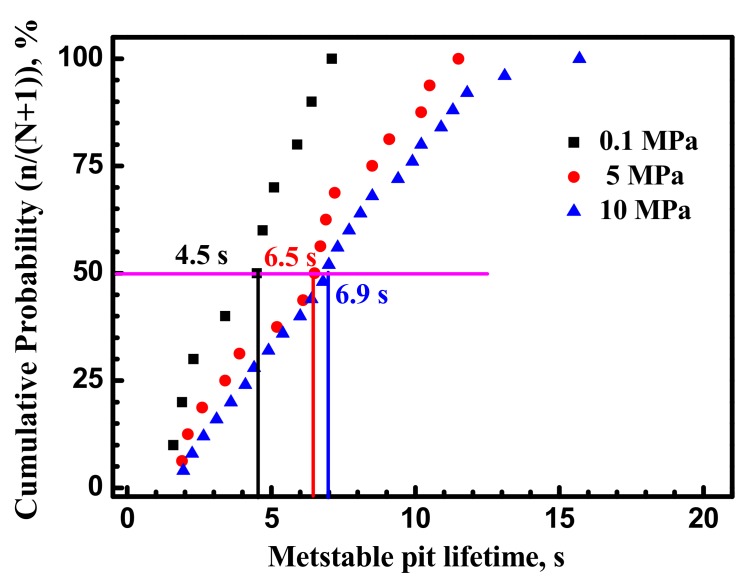
Cumulative distribution of metastable pits lifetime for the X70 steel at various hydrostatic pressures and −100 mV vs. Ag/AgCl electrode.

Lifetime is defined as the time between when metstable current transients begin to undergo repassivation initiation. Transient lifetime of metastable pit events at 0.1 MPa, 5 MPa and 10 MPa is plotted in Figure 6. In this figure, the cumulative probability of the metstable pit lifetime was calculated as n/(N + 1) using a mean rank method [27], where N is the total number of pits and n is the order in the total number. There is an increase in metastable pitting lifetime with hydrostatic pressure. Long lifetime of metastable pitting means that inclusions are further dissolved and the repassivation is weak.

**Figure 7:**
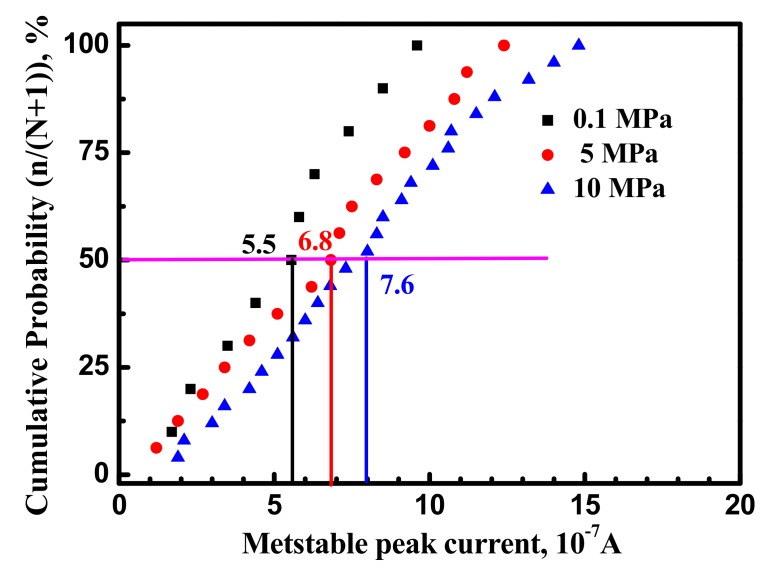
Cumulative distribution of metastable pits i_peak_ for the X70 steel at various hydrostatic pressure and −100 mV.

Peak current (i_peak_) is defined as, i_peak_ = i_max_ − i_bc_. Figure 7 shows the value of peak current of metastable transients of the X70 steel at various hydrostatic pressure and −100 mV. The peak current (i_peak_) will increase with hydrostatic pressure. X70 steel at 0.1 MPa, 5 MPa and 10 MPa has the peak current (i_peak_) values of 55.4 nA, 68.1 nA and 76.7 nA. Higher peak current indicated that many more active atoms were dissolved.

It is assumed that metastable pitting is hemispherical. Following Pistorius and Burstein [12,13], metastable pitting radius is calculated using the Faraday Equation [28,29]:

(1) rpit=[(3Z2πnFρ)∫tgtp(Ipeak−Iorg)dt]13

Here, *Z* is the mean molecular weight of 55.2 mg·mol^−1^, *ρ* is the mean alloy density of 7.9 mg·cm^−3^ and *F* is the Faraday’s constant. For the studied X70 steel it was assumed that the Fe element is oxidized to Fe^2+^ during corrosion. Hence, the oxidation state of cations (n) is considered to be 2.

**Figure 8:**
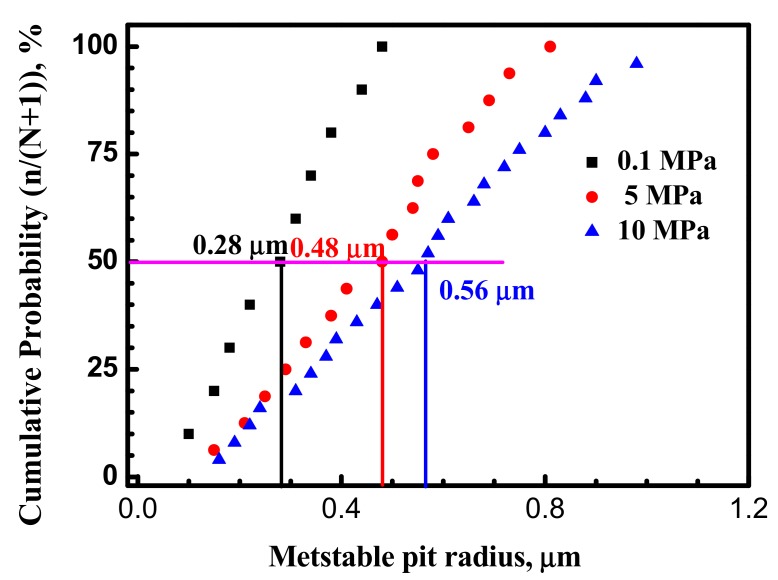
Cumulative distribution of metastable pits radius for the X70 steel at various hydrostatic pressure and −100 mV vs. Ag/AgCl electrode.

Figure 8 shows calculated radius of metastable pits using Equation (1) for the X70 steel at various hydrostatic pressure. Comparing the value of metastable pits radius, it can be seen that the metastable pits radius at 10 MPa is larger than at 5 MPa and at 0.1 MPa.

**Figure 9:**
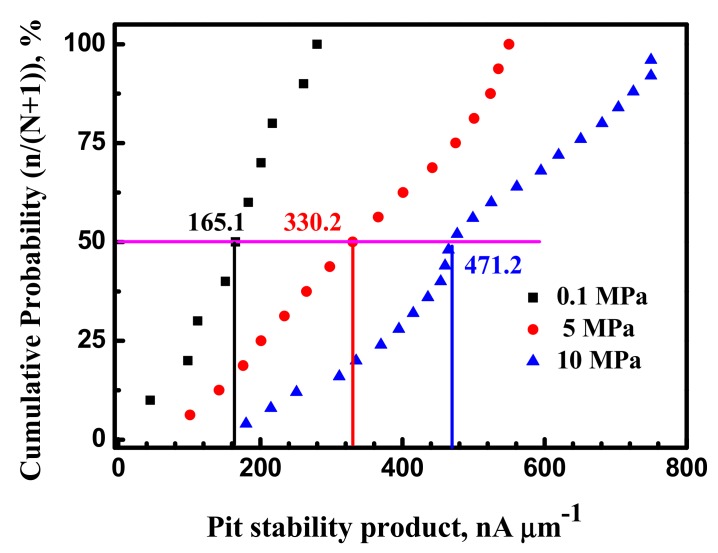
Cumulative distribution of metastable pits stability for the X70 steel at various hydrostatic pressure and −100 mV vs. Ag/AgCl electrode.

Pit propagation is based on the diffusion criterion proposed by Pistorius and Burstein [1]. It is widely believed that stable pitting can transform from metastable pitting if the current density of metastable reaches the minimum and metastable pitting size is beyond the threshold value, i_peak_ × r_pit_ [15]. A metastable pit, which survived from the nucleation stage, grows unstably in the diffusion-controlled regime [13]. Rupture of corrosion products or inclusion dissolution promote anode dilution below the threshold current value, followed by repassivation, stopping metastable pit growth (for example type B inclusion). If the surface corrosion products rupture, promoting anode dilution above the critical current value, pit stabilization occurs (for example type C inclusion). In the present work, the i_peak_ × r_pit_ values under various hydrostatic pressure are calculated, shown in Figure 9. Comparing the average value of i_peak_ × r_pit_, it is evident that higher hydrostatic pressure results in higher pits stability. This means that hydrostatic pressure promotes metastable pitting propagation.

**Figure 10:**
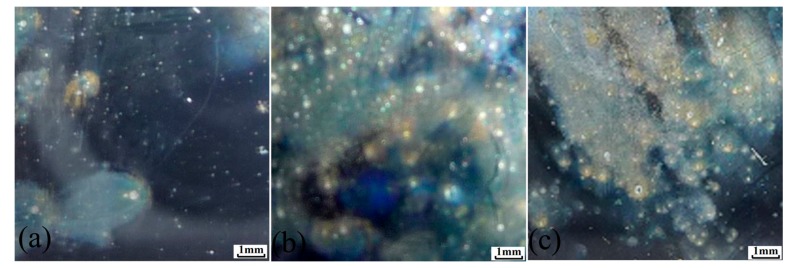
Corrosion macroscopic morphology of the X70 steel after immersion for 30 min in 0.1 mol/L NaCl solution at: (**a**) 0.1 MPa; (**b**) 5 MPa and (**c**) 10 MPa.

**Figure 11:**
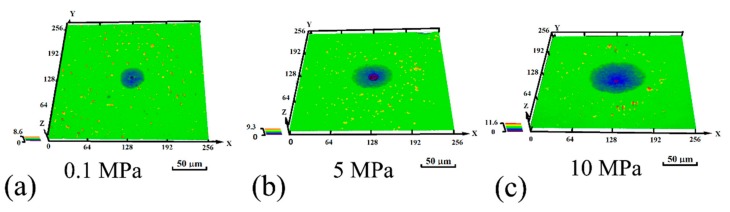
Typical 3D morphology of corrosion pits after immersion for 30 min in 0.1 mol/L NaCl solution at: (**a**) 0.1 MPa; (**b**) 5 MPa and (**c**) 10 MPa.

**Figure 12:**
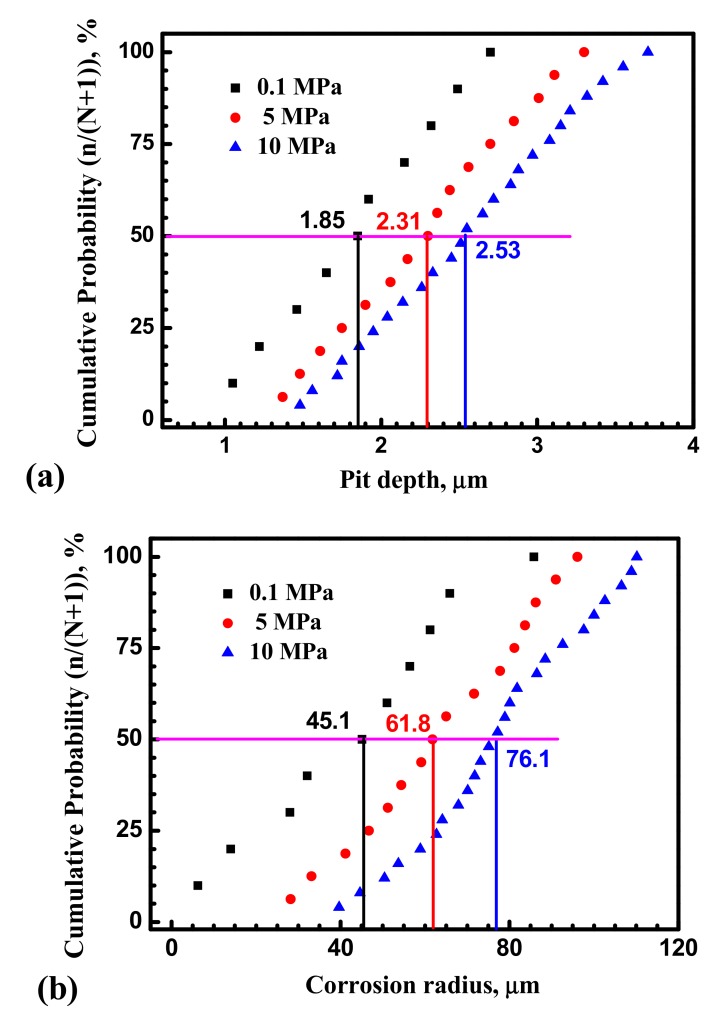
The 3D sizes of corrosion pits of the X70 steel at 0.1 MPa, 5 MPa and 10 MPa after 30 min immersion in 0.1 mol/L NaCl solution: (**a**) Pit depth; (**b**) Corrosion radius.

Corrosion morphology of the X70 steel immersed for 30 min in 0.1 mol/L NaCl solution at 0.1 MPa, 5 MPa and 10 MPa is shown in Figure 10. Confocal microscopy images of typical pits morphology of the X70 steel at different hydrostatic pressure are shown in Figure 11. Statistical distribution of the 3D pit size is plotted in Figure 12.

By comparing corrosion morphology at different hydrostatic pressure, it is found that the X70 steel corrosion begins with generation of corrosion pitting and corroded area at 10 MPa is larger than at 5 MPa and 0.1 MPa. The number of pitting initiation sites at 10 MPa is much larger than at 5 MPa and 0.1 MPa. This implies that the pitting initiation is accelerated by hydrostatic pressure. This result is consistent with the value of metastable pits stability for the X70 steel. Hydrostatic pressure promotes pitting initiation and propagation.

## 4. Discussion

### 4.1. Hydrostatic Pressure Effect on Metastable Pit Initiation

Previous researchers analyzed metastable pit by counting the generation frequency of transient current peaks. Williams [30,31] reported a linear relationship between metastable and stable pitting.

Figure 3 and Figure 5 show a larger number of metastable pits with hydrostatic pressure. Relatively higher frequency of metastable pit formation could be explained based on the ability of inclusions to dissolve and form weaker passive film [13,14]. Beccaria and Sun [21,22] reported that high hydrostatic pressure can promote the adsorption of Cl^−^ on metal surface, especially in inclusion site. Increased Cl^−^ can enhance dissolution of the inclusions. In addition, weaker passive film can be formed at inclusion position with increasing Cl^−^ content. Thus, many more nucleation points would be generated at inclusions site. The frequency of metastable pitting initiation is increased with hydrostatic pressure. As shown in Figure 10, the number of pits on the X70 steel surface is significantly higher at 10 MPa.

### 4.2. Hydrostatic Pressure Effect on Metastable Pit Growth

Metastable pitting growth rate is affected by the environment around pitting. Passive film structure and stress conditions around the pit are quite complex [6,15]. Figure 6 and Figure 7 show that the metastable pits developed at higher hydrostatic pressure (10 MPa) are accompanied with higher peak current and longer lifetime. It can be concluded that higher hydrostatic pressure promotes anodic dissolution around and inside the pit site in the X70 steel. Figure 8 clearly shows that higher hydrostatic pressure increases calculated radius of metastable pitting. According to the results, metastable pit current is a measure of its growth rate. Similar to corrosion rate of steel, higher current peak and longer lifetime at higher hydrostatic pressure indicate higher corrosion rate.

Previous work found that localized stress around pitting can significantly affect pit growth rate [23]. Hydrostatic pressure can affect the electrochemical activity of deformed metal. According to Gutman’s theory [10,11], the anodic dissolution non-equilibrium kinetic equation can be defined as:

(2) $$i_{p}=i_{a}\exp\frac{\Delta pV_{m}}{RT}$$

Here, *i_p_* is the anodic dissolution current, *i_a_* is the anodic current of an unstressed sample, *p* is the spherical part of macroscopic stress tensor (i.e., hydrostatic pressure) depending on the applied load and *V_m_* is the molar volume of the substance [11,32].

According to Yang et al., stress concentration is induced by corrosion pitting during loading with hydrostatic pressure. Then corrosion will preferentially dissolve the pitting site. At the same time, stress concentration at corrosion pits increases with hydrostatic pressure [10]. It can further promote steel anodic activity around the pit.

**Figure 13:**
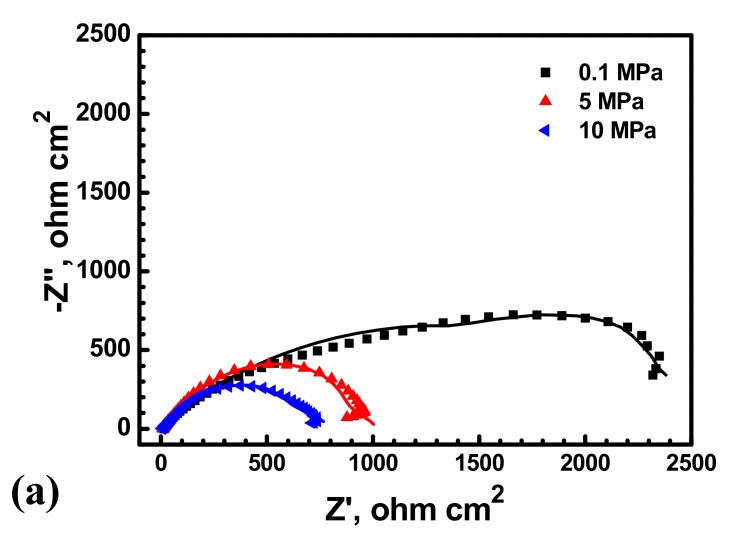

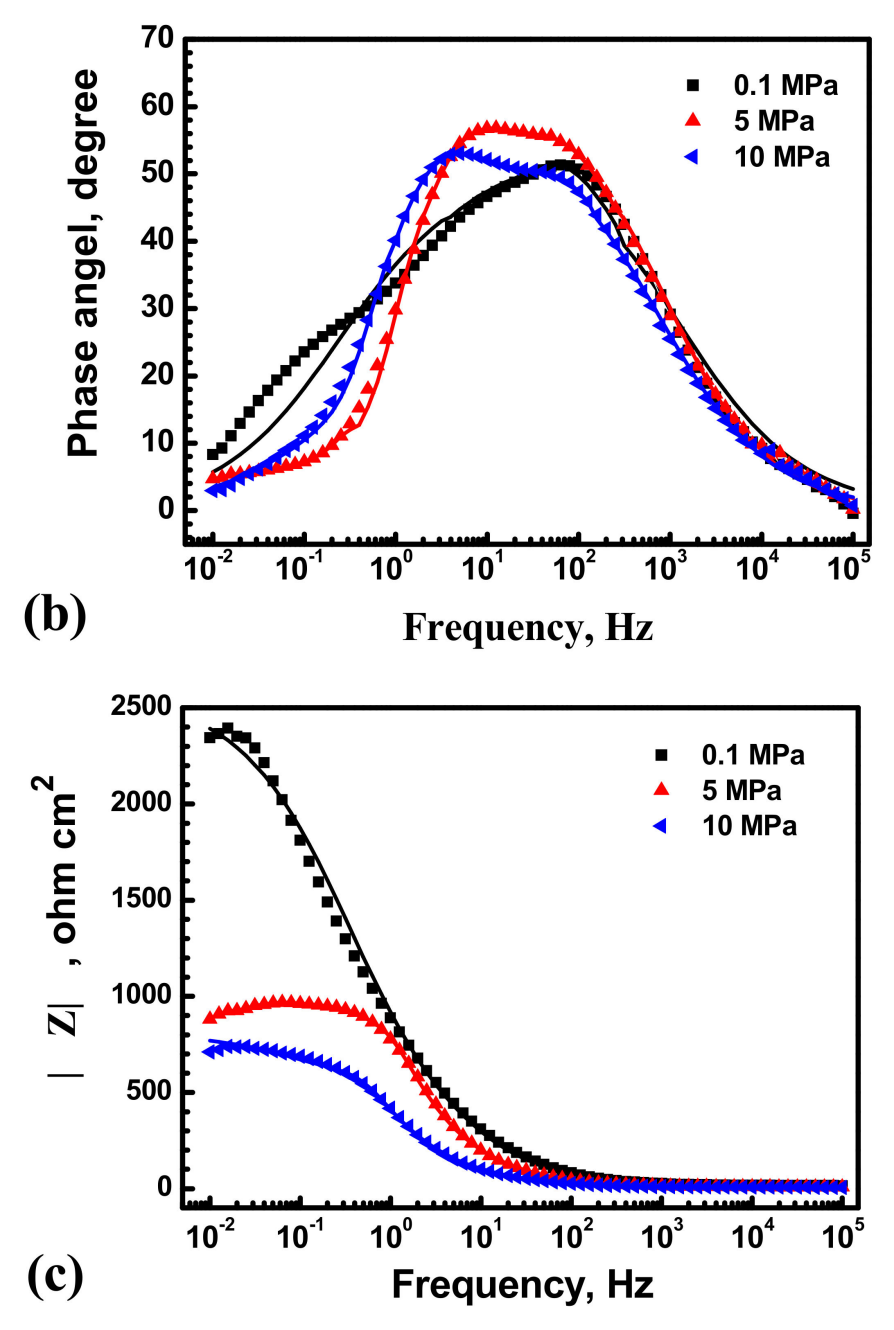
Impedance spectra of the X70 steel measured at the open circuit potential in 0.1 mol/L NaCl solution at 0.1 MPa, 5 MPa and 10 MPa. Solid lines represent fitted results. (**a**) Nyquist plot; (**b**) Bode-Phase angle plot; (**c**) Bode-impedance plot.

In addition, Figure 13 shows the impedance spectra at different hydrostatic pressure. As shown in Figure 13, it is remarkable that the diameters of semicircles decrease with hydrostatic pressure, the impedance markedly decrease with hydrostatic pressure at low frequencies region indicating that electrochemical process is accelerated by hydrostatic pressure after 30 min immersion. Generally, for EIS measured on bare low alloy steel samples, the charge transfer resistance is the only time constant [33]. Thus, hydrostatic pressure decreases the charge transfer resistance and increases the dissolution rate within the cavities. As shown in Figure 12, the radius of the X70 steel surface pits is significantly larger at 10 MPa.

### 4.3. Hydrostatic Pressure Effect on Metastable Pitting Stabilization

Higher hydrostatic pressure would result in higher i×r value, as shown in Figure 10. It can be concluded that metastable pitting developed at higher hydrostatic pressure of 10 MPa results in much higher stability products than at lower hydrostatic pressure of 0.1 MPa. Therefore, at 10 MPa, metastable pitting stabilization is easier than at 0.1 MPa.

Williams et al. [30,31] reported that pitting corrosion susceptibility is directly related to the frequency of metastable pitting formation. Pitting resistance dependence on the frequency of metastable pitting formation can be expressed as:

(3) $$\Lambda =\lambda \exp(-\mu \tau_{c})$$

Here, Λ is the rate of formation of stable pitting, *λ* is the rate of formation of metastable pitting, *μ* is the repassivation probability of metastable pitting, and *τ*_c_ is the critical time when pits are considered stable [34,35].

Metastable pit frequency results (Figure 6) confirm that higher hydrostatic pressure significantly improves the frequency of metastable pitting formation. It can be concluded that increasing hydrostatic pressure would lead to decreased corrosion resistance of the X70 steel. The frequency of stable pit formation increases with hydrostatic pressure.

Besides, individual metastable current transients indicated that hydrostatic pressure speeds up dissolution kinetics and promotes pitting stabilization. Thus, hydrostatic pressure would improve pitting corrosion susceptibility of the X70 steel. This is consistent with the result of corrosion tests, which revealed that hydrostatic pressure increases the number of pits in Figure 12. This was verified by evaluating metastable pitting to predict pitting corrosion resistance.

## 5. Conclusions

In this paper, the hydrostatic pressure effects on pitting initiation, metastable pitting and propagation in X70 steel have been investigated by evaluating metastable pitting events and immersion tests. The results could be summarized as follows:

(1) Potentiodynamic measurements indicated that increasing hydrostatic pressure decreases the breakdown potential and leads to reduced transpassivity region.
(2) Hydrostatic pressure can promote the adsorption of Cl^−^ on metal surface. Potentiostatic measurement indicated that the rate of metastable pit formation in the X70 steel increased with hydrostatic pressure, indicating that the pitting generation rate was increased. EIS results indicate Hydrostatic pressure decreases the charge transfer resistance and increases the dissolution rate within the cavities. The results also revealed that increasing hydrostatic pressure leads to an increase in the average values of metastable pitting peak current, pit radius and pit lifetime. Hydrostatic pressure improves the probability of metastable pits transition to stability. This means that hydrostatic pressure promotes metastable pitting initiation and propagation.
(3) Metastable pit measurement and corrosion tests both indicated that pitting initiation and propagation are accelerated by hydrostatic pressure. Result validity is verified by evaluating metastable pitting to predict pitting corrosion resistance.

## Figures and Tables

**Table 1:** Chemical composition of the X70 steel specimens (wt %).

C	Si	Mn	Cr	Ni	Ti	V	Nb	Others	Fe
0.066	0.29	1.39	0.032	0.20	0.015	0.037	0.056	0.300	Bal.
